# Nomogram prediction of surgical site infection of HIV-infected patients following orthopedic surgery: a retrospective study

**DOI:** 10.1186/s12879-020-05613-3

**Published:** 2020-11-26

**Authors:** Rui Ma, Jie He, Biao Xu, Changsong Zhao, Yao Zhang, Xin Li, Sheng Sun, Qiang Zhang

**Affiliations:** Department of Orthopaedics, Beijing Ditan Hospital, Capital Medical University, No. 8 Jingshun East Street, Chaoyang District, Beijing, 100015 China

**Keywords:** Nomogram, Surgical site infection, Orthopedic, HIV, CD4, Erythrocyte sedimentation rate

## Abstract

**Background:**

Surgical site infection (SSI) is a devastating complication of orthopedic surgery, related with increased morbidity and mortality. This study was performed with the aim to compare the SSI rate in human immunodeficiency virus HIV-positive patients, to identify other risk factors for SSI and to establish a nomogram model to predict the risk of SSI.

**Methods:**

A total of 101 HIV-positive individuals following orthopedic surgery patients admitted to Beijing Ditan Hospital. Their characteristics were gathered. The univariate and multiple logistic regression analysis were performed to explore the risk factors of SSI. And the Nomogram prediction model was constructed and verified.

**Results:**

The independent predictive factors of SSI included CD4 (Odds ratio [OR], 0.041; *P* = 0.040), erythrocyte sedimentation rate (ESR) (OR, 89.773; *P* = 0.030), and procalcitonin (PCT) (OR, 220.746; *P* = 0.006). The scoring nomogram model was as follows: Logit (SSI) = − 2.63589–0.00314*CD4 < 430.75 = 1) + 0.04695*(ESR < 17.46 = 1) + 2.93694*(PCT < 0.22 = 1). The area under the Receiver Operating Characteristic (ROC) curve was 0.946. The cutoff score was − 2.1026 with a sensitivity of 93.33% and a specificity of 84.88%.

**Conclusions:**

CD4, ESR, PCT might affect the occurrence of SSI after orthopedic surgery. The nomogram model constructed in this study is helpful for predicting the probability of SSI.

## Introduction

Surgical site infection (SSI) is an adverse complication of orthopedic surgery and can increase the risk of readmission [1, 2]. Moreover, SSI often caused poor prognosis, decreased quality of life and the possibility of reoperation [3, 4]. It is well known that HIV patients are more likely to develop SSI than those who are not infected with HIV due to their dramatically decreased CD4 cell count and weak immune resistance [5]. With the extension of HIV infection time, the probability of opportunistic infection increases greatly, and postoperative orthopedic incision is prone to infection [6].

In clinical orthopedic surgery, internal fixation and implant devices are often used. Due to the body’s autoimmunity rejection of foreign objects, the chance of postoperative wound infection is greatly increased [7]. Internal fixation and implant devices are kept in the body for a long time, creating space and attachments for the growth of pathogens. Therefore, not only does the incidence of SSI increase in the early stage of orthopedic surgery, but it also increases the risk of infection in the later stage. If the internal fixation and implant devices become infected, the body must be treated with antibiotics for a long time, and the internal fixation and implant devices should be removed or replaced as soon as possible. This will greatly increase the medical costs of patients [8]. Currently, there are few quantitative studies and prediction models which show that HIV virus and reduced CD4 count increase the risk of infection with internal fixation and implant devices during orthopedic surgery [9, 10]. According to WHO guidelines on infection prevention and control, penicillin should be used for prevention as soon as 1 hour after orthopedic surgery, and the duration should not exceed 24 h [11]. However, clinicians generally expect the incidence of postoperative SSI to be less than 2% [12].

Although there are currently surgical guidelines for internal fixation and implant devices, these are based only on data from non-HIV patients. Specific orthopedic guidelines for HIV patients do not yet exist. Therefore, it is necessary for orthopedic surgeons in hospitals with infectious diseases to formulate corresponding operational guidelines. The purpose of this study was to develop a Nomogram prediction model for the incidence of SSI in patients with HIV who underwent orthopedic internal fixation and implant surgery.

## Materials and methods

### Patients and ethics

**Fig. 1:**
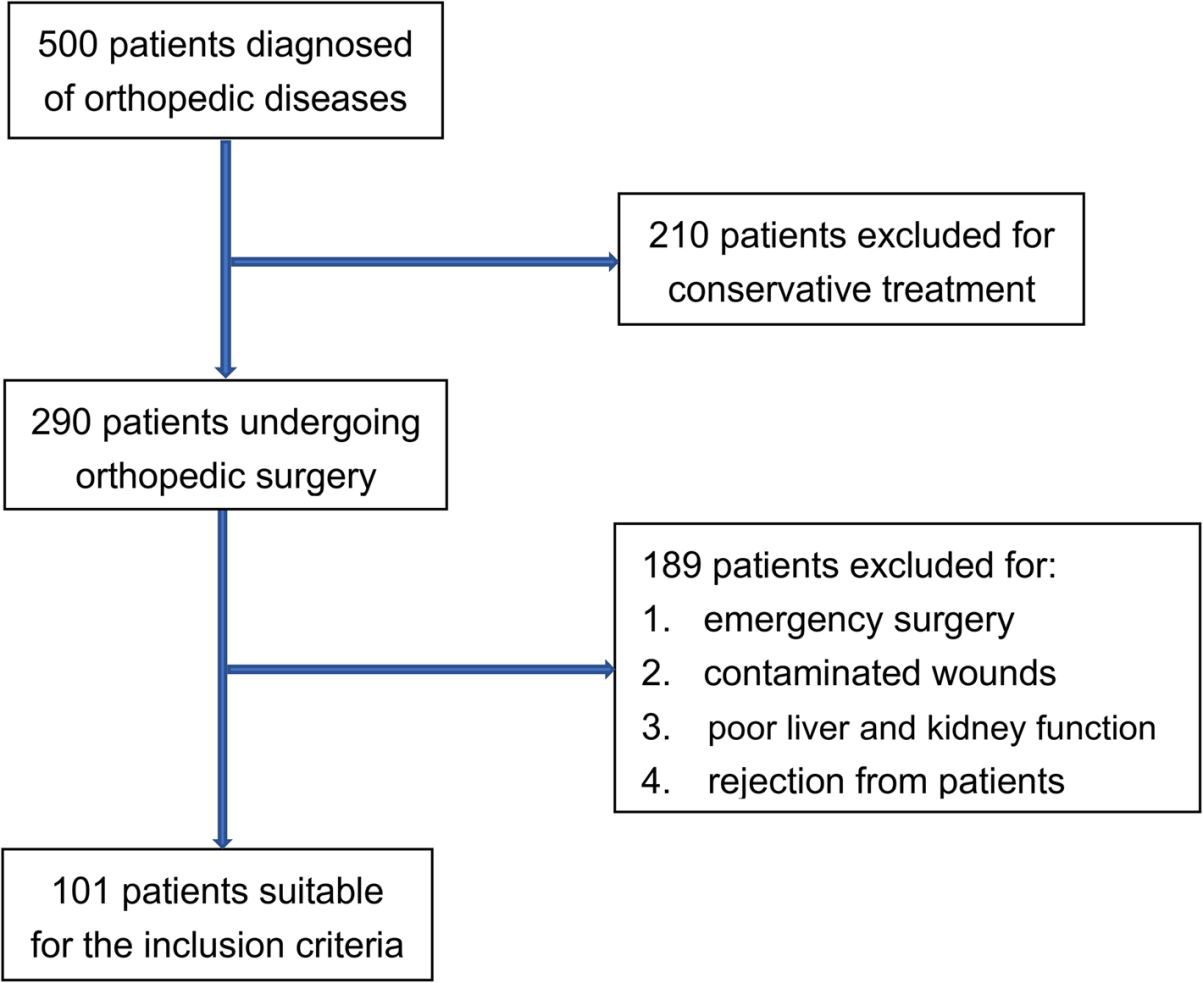
Selection process for patients suitable for the inclusion criteria

This study is a retrospective study. A total of 101 patients with HIV-positive fractures were enrolled in our department. We have referred to this document and calculated the sample size required for this study [13]. The results are as follows. Therefore, 101 HIV-positive patients can satisfy this study. Follow up by phone and notify the patient of the infection. Observe whether the wound is infected according to the guidelines for infection at the surgical site. The follow-up time was 1 year. The follow-up rate in this study was 100%. These patients underwent orthopedic surgery between April 2018 and August 2019 in the Beijing Ditan Hospital. Antibiotic dosage is 8 g/day cefradine. The surgeries were performed by professors in our orthopedic department and all surgeons have performed orthopedic surgery for more than 500 cases. Patients with open fractures require emergency surgery and contaminated wounds were excluded. Patients with poor liver and kidney function were excluded for the high risk of postoperative complications and death (Fig. 1). Laboratory examination of abnormal liver function is mainly manifested as AST and/or ALT > 40 U/L. The criterion of abnormal kidney function is mainly manifested as creatinine >115umol/L(male) or > 97 umol/L(female), urea nitrogen> 7.1umol/L.

Preoperative treatment including intravenous or oral amino acids, albumin injection, and thymopentin were administered as routine nutritional supplementation to improve nutrition and hypoalbuminemia. Infusions of red blood cell suspension and/or plasma were administered as necessary. A second-generation cephalosporin was routinely administered within 2 h of the skin incision for all patients. In patients with allergies to cephalosporins, clindamycin was an acceptable alternative. Antibiotics were administered for 3 days and then continued as needed based on the incision condition, body temperature, white blood cell count, erythrocyte sedimentation rate, C-reactive protein level, and bacterial culture. Low molecular weight heparin and venous pressure pump were utilized postoperatively to prevent DVT(Deep venous thrombosis)s [14].

This study was approved by the Ethical Committee of the Beijing Ditan Hospital. And the written informed consent was obtained from the all patients.

### The diagnosis of SSI

The diagnosis of SSI was confirmed by postoperative incision observation and imaging examination. All participants underwent clean implant orthopaedic surgery and the incidence of SSI was evaluated. The classification of SSI was based on Systematic Literature Review on the Management of Surgical Site Infections (June 2018) published by the American Academy of Orthopaedic Surgeons (AAOS) [15]. The SSI criteria used were those established by the Centers for disease control and prevention guideline for the prevention of surgical site infection [16].

### The clinical variables

We collect clinical data through patient medical records and follow-up. All the laboratory results were baseline data. Select variables known to be associated with SSI were chosen for inclusion in the nomogram including sex, age, basic disease [17] (that is hypertension, diabetes, coronary heart disease, and so on), surgical methods (open or minimally surgical technique), operation time, CD4 of peripheral blood, HIVRNA, creatinine, albumin, C-reactive protein (CRP), Globulin, ESR (Erythrocyte sedimentation rate), PCT(Procalcitonin), D-dimmer. In this study, the mean value was used as the critical value. All laboratory tests are performed the day before surgery.

The basic disease refers to the long-term/chronic coexisting disease that affects basic metabolism, immune function, and vital organs that the patient itself has. The basic diseases mainly include the following three types of diseases: basic metabolic disorders, low immune function and major chronic wasting diseases.

### Statistics

SPSS 25.0 (IBM Corp., Armonk, NY, USA) statistical software was used to analyze the data. Quantitative variables were shown as the mean ± standard deviation (SD). Qualitative variables were described by absolute frequencies and percentages. The descriptive statistical method of mean ± SD is adopted in the measurement data, and the frequency and percentage description method are used in the counting data.

For continuous variables, the rank sum test of Kruskal Wallis was used. For counting variables with theoretical number < 10, the Fisher exact probability test was used. Risk factors were screened by univariate and multiple logistics regression. Perioperative clinical characteristics associated with postoperative (incisional and organ/space) SSI were analyzed by univariate analyses.

### Construction of the nomogram prediction model

The nomogram was established with R (http://www.R-project.org) and Empower Stats (X&Y Solutions Inc., Boston, MA, USA) software. In the single and multiple logistic regression model, indicators with P less than 0.05 are included in the nomogram model. Based on the total score obtained from the analysis results, the reliability of the risk assessment model based on the total score was evaluated by ROC analysis method, and a post-operation SSI risks assessment model was established.

## Results

### Associations between characteristics and SSI

The incidence of SSI in this research was 14.85%. Table 1 summarized the associations between potentially risk factors and the SSI according to the rank sum test of Kruskal Wallis and the Fisher exact probability test. Among the individuals, the basic disease (*P* = 0.020), operation time (*P* = 0.005) and CD4 (*P* = 0.044), CRP (*P* = 0.003), ESR(*P* < 0.001), PCT (*P* < 0.001), D-dimmer (*P* < 0.001) were markedly related to the SSI (Table 1). The incidence of SSI in the patients with high HIVRNA expression was 17.39%, which is higher than the patients with low HIVRNA expression (14.10%) (*P* > 0.05) (Table 1).

**Table 1:** The association between clinical and pathological characteristics and SSI

Characteristics	SSI		*P*-value
	No (*n* = 86)	Yes (*n* = 15)	
Sex			0.463
Male	83 (96.5%)	15 (100.0%)	
Female	3 (3.5%)	0 (0.0%)	
Age	42.9 ± 14.1	39.7 ± 12.5	0.406
Basic disease			0.020*
Yes	54 (62.8%)	14 (93.3%)	
No	32 (37.2%)	1 (6.7%)	
Surgical methods			0.107
Microsurgery	64 (74.4%)	14 (93.3%)	
Open surgery	22 (25.6%)	1 (6.7%)	
Operation time			0.005*
1–2 h	45 (52.3%)	2 (13.3%)	
2–3 h	18 (20.9%)	3 (20.0%)	
≥ 3 h	23 (26.7%)	10 (66.7%)	
CD4	449.2 ± 229.3	324.9 ± 132.4	0.044*
HIVRNA			0.697
Low	67 (77.9%)	11 (73.3%)	
High	19 (22.1%)	4 (26.7%)	
Creatinine	70.9 ± 14.3	64.4 ± 12.0	0.103
CRP	9.7 ± 19.9	27.6 ± 26.2	0.003*
Albumin	44.5 ± 7.0	42.3 ± 5.1	0.245
Globulin	28.9 ± 4.5	31.6 ± 6.2	0.047*
ESR	14.5 ± 15.4	34.2 ± 21.1	< 0.001*
PCT	0.1 ± 0.2	0.9 ± 0.7	< 0.001*
D-dimmer	1.7 ± 3.0	7.1 ± 10.3	< 0.001*

The data was presented with Mean + SD/N (%). For continuous variables, the rank sum test of Kruskal Wallis was used. For counting variables with theoretical number < 10, the Fisher exact probability test was used. *P-value≤0.05

### The risk factors of SSI based on univariate logistic regression analysis

Table 2 presents the univariate OR and 95% confidence intervals (95%CI) for SSI. The OR for SSI was 0.121 (95% CI, 0.015–0.960, *P* = 0.046) in the group without basic disease compared with patients with basic disease. For SSI, long operation time had higher OR of 9.783 (95% CI, 1.977–48.412, *P* = 0.005) than subjects with 1–2 h’ operation time. Subjects who had high CD4, had obviously lower incidence of SSI than subjects who had low CD4, and the OR is 0.239 (95% CI, 0.063–0.906, *P* = 0.035). Moreover, the higher risk of SSI was often accompanied by the higher levels of CRP (OR, 7.714; *P* = 0.001), ESR (OR, 11.636; *P* = 0.000), PCT (OR, 44.550; *P* = 0.000), D-dimmer (OR, 4.913; *P* = 0.008) (Table 2).

**Table 2:** Correlative parameters’ effect on SSI based on univariate logistic regression analysis

Characteristics			SSI		
			OR	95% CI	P
Sex	Male	98	1		0.999
	Female	3	0.000	0.000–0.000	
Age	≤65	91	1		0.652
	> 65	10	0.611	0.072–5.210	
Basic disease	Yes	68	1		0.046*
	No	33	0.121	0.015–0.960	
Surgical methods	Microsurgery	78	1		0.140
	Open surgery	23	0.208	0.026–1.673	
Operation time	1–2 h	47	1		
	2–3 h	21	3.750	0.577–24.351	0.166
	≥3 h	33	9.783	1.977–48.412	0.005*
CD4	Low	54	1		0.035*
	High	47	0.239	0.063–0.906	
HIVRNA	Low	78	1		0.296
	High	23	1.889	0.573–6.225	
Creatinine	Low	54	1		0.583
	High	47	0.732	0.240–2.234	
CRP	Low	78	1		0.001*
	High	23	7.714	2.368–25.131	
Albumin	Low	56	1		0.347
	High	45	0.575	0.181–1.823	
Globulin	Low	52	1		0.339
	High	49	1.725	0.565–5.269	
ESR	Low	67	1		0.000*
	High	34	11.636	3.002–45.098	
PCT	Low	85	1		0.000*
	High	16	44.550	10.368–191.417	
D-dimmer	Low	81	1		0.008*
	High	20	4.913	1.520–15.887	

*OR* odds ratio, *95% CI* 95% confidence interval. * *P* < 0.05

### The independent risk factors for SSI based on multivariate logistic regression

In order to effectively control the influence of confounding factors, all risk factors were incorporated into the multivariate logistic regression model simultaneously, which can also predict the most independent risk characteristic. Multivariate logistic regression analysis showed that risk factors indicating overall postoperative SSI included CD4 < 430.75/ul(OR[CD4 > 430.75/ul VS. CD4 < 430.75/ul]: 0.041; 95% CI:0.002–0.868; *P* < 0.040), ESR > 17.46(OR[ESR > 17.46 VS ESR < 17.46]: 89.773; 95% CI: 1.551–5195.833; *P* = 0.030), PCT > 0.22(OR[PCT > 0.22 VS PCT < 0.22]: 220.746; 95% CI: 4.829–10,091.115; *P* = 0.006) (Table 3).

**Table 3:** Correlative genes’ effect on SSI based on multiple logistic regression analysis

Characteristics	SSI		
	OR	95% CI	P
Sex	0.000	0.000–0.000	0.999
Age	0.018	0.000–1.619	0.080
Basic disease	0.044	0.000–5.403	0.203
Surgical methods	0.267	0.008–8.516	0.455
Operation time	1.141	0.245–5.313	0.867
CD4	0.041	0.002–0.868	0.040*
HIVRNA	0.085	0.003–2.256	0.140
Creatinine	30.768	0.744–1272.930	0.071
CRP	2.278	0.116–44.604	0.588
Albumin	1.269	0.088–18.394	0.862
Globulin	0.456	0.039–5.317	0.531
ESR	89.773	1.551–5195.833	0.030*
PCT	220.746	4.829–10,091.115	0.006*
D-dimmer	11.780	0.204–679.695	0.233

*OR* odds ratio, *95% CI* 95% confidence interval. *P < 0.05

### The nomogram and its predictive performance

**Fig. 2:**
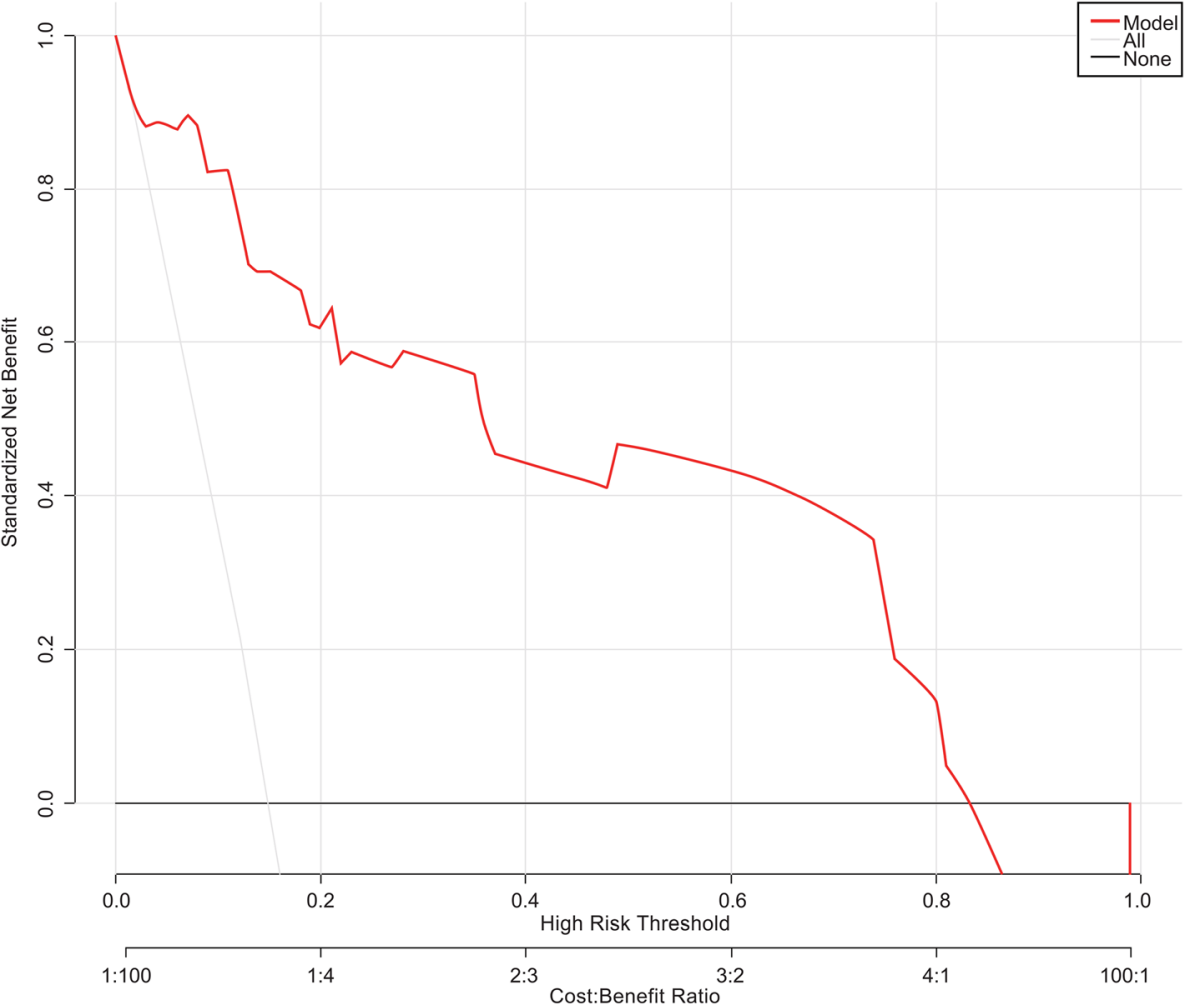
The standardized net benefit, high risk threshold, and benefit ratio of model

**Fig. 3:**
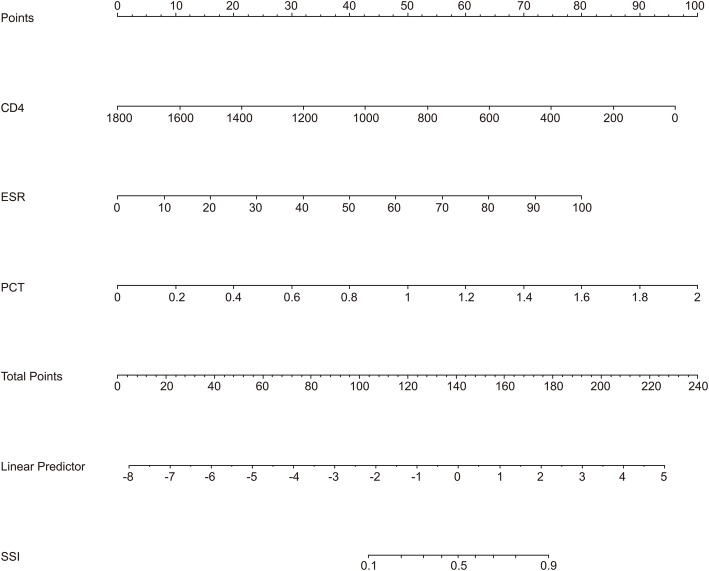
Nomogram for estimating the risk of SSI in patients. SSI: Surgical site infection; ESR: Erythrocyte sedimentation rate; PCT:Procalcitonin

The regression co-effificients from logistic model were used to construct the model for estimation of SSI risk. The standardized net benefit, high risk threshold, and benefit ratio of model were manifested (Fig. 2). The scoring model was as follows: Logit(SSI) = − 2.63589–0.00314*CD4 < 430.75/ul = 1) + 0.04695*(ESR < 17.46 = 1) + 2.93694*(PCT < 0.22 = 1), which was also presented visually (Fig. 3).

### The verification of sensitivity and specificity of nomogram model

**Fig. 4:**
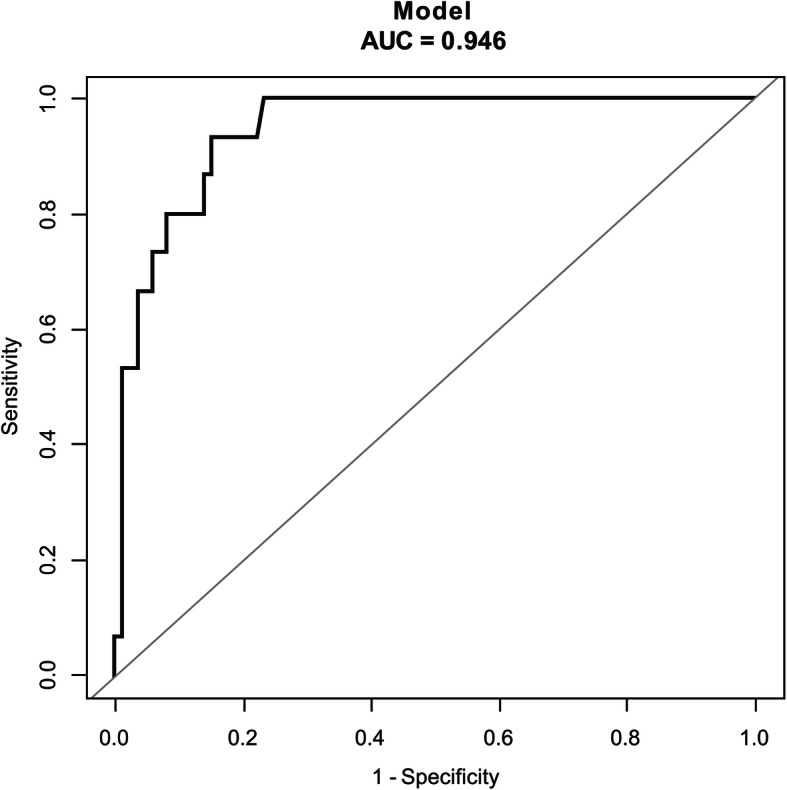
The ROC of the model from observed data (nomogram) was 0.946. The cutoff score was − 2.1026 with a sensitivity of 93.33% and a specificity of 84.88%. ROC: Receiver Operating Characteristic; AUC: Area under curve

The performance of the nomogram was measured by ROC curves and the area under curve (AUC) was 0.946 (95% CI 0.901–0.991) in the model from observed data. The cut-off score was − 2.1026 with a sensitivity of 93.33% and a specificity of 84.88% (Fig. 4).

## Discussion

In this study, we sought to identify the independent risk factors in adult patients susceptible to SSI after instrumented fusion surgery for orthopedic diseases. We analyzed the clinical data of 101 patients who underwent orthopedic surgery in Beijing Ditan Hospital from April 20 to August 2019. According to whether patients had SSI, they were divided into two groups. Preoperative CD4 T cells, presence or absence of opportunistic infection, and organ dysfunction were used to identify whether there was SSI in the patients [16].

According to the results, SSI is mainly related to factors such as basic disease, operation time and CD4, CRP, ESR, PCT, and D-dimmer. Multivariate logistic regression analysis showed that risk factors indicating overall postoperative SSI included CD4 < 430.75/ul, ESR > 17.46, and PCT > 0.22. The scoring model was as follows:Logit(SSI) = − 2.63589–0.00314*CD4 < 430.75/ul = 1) + 0.04695*(ESR < 17.46 = 1) + 2.93694*(PCT < 0.22 = 1). The model consisting of risk factors might be used to accurately assess whether a patient has SSI.

The results of this study were slightly different from the results reported in the current literature. Firstly, the subjects in the former study [18] were all a type of incision, but the results of this study did not include the relevant factors of the incision category. Moreover, targeted application of nutritional support treatment before surgery, so that patients better tolerate surgery, so albumin and hemoglobin have not become a risk factor affecting surgical site infection [19]. Pay attention to perioperative nutritional support and immune reconstitution in HIV-positive patients, correct treatment of hypoproteinemia and anemia, and infusion of plasma or suspended red blood cells if necessary [19]. Older patients should be paid more attention to the adjustment of this situation. The results of this study showed that the SSI rate was 14.85%, further confirming the significance of standardized treatment during perioperative period.

The CD4+ T count were significantly associated with the incidence of SSI in patients [20]. Reported in the literature, CD4 T lymphocyte count< 200/ul and viral load> 500,000 copies/ul, the incidence of postoperative incision infection wound would increase [21]. Therefore, in order to prevent surgical site infection, it is necessary to control the patient’s CD4 count and improve the patient’s immunity status [22]. At the same time, apply clinical classification of HIV System, to assess the safety of surgery, for patients with CD4 T lymphocyte count higher than grade 2, careful consideration of surgery, it is best to adjust the CD4 T lymphocyte count to a higher level after elective surgery, it is best to improve the perioperative adjuvant treatment after elective surgery [23].

However, the choice of whether to operate and the timing of surgery cannot be completely dependent on the CD4 T count, and it is necessary to determine the tolerance to surgery in combination with the patient’s general condition, such as ESR and PCT.

The ESR level also affected the incidence of surgical site infections in orthopedic patients with HIV-positive patients. HIV patients have low or even reduced immunity and a higher risk of opportunistic infections [24]. More and more complex internal fixations were likely to cause infection of the incision, and the disturbance to the physiological function of the body is also large, which could active the inflammatory system [25]. When the acute inflammation occurred, blood acutephase reactant increased rapidly, including α-antirypsin, α2-mactoglobulin, C reactive protein, haptoglobin, transferrin, fibrinogen, etc. [26]. The main reason is that the above components, which were released increasingly, could promote the rouley-like aggregation of red blood cells to a greater or lesser extent [27]. The rapid increase of ESR could be seen in 2–3 days after the occurrence of inflammation. Therefore, under the premise of following the basic principles of orthopedic surgery, surgery should use precise incision, fixation tendencies, such as the selection of simple and effective fixed equipment. The ESR level should be detected timely to estimate the incidence of SSI.

PCT is a protein, which increased in the SSI patients (OR = 220.746, *P* < 0.05). Its levels in the plasma rose in severe bacterial, fungal and parasitic infections as well as sepsis [28]. Bacterial endotoxin played an important role in the induction process [29]. PCT was a parameter for the diagnosis and monitoring of bacterial inflammatory disease infections. Elevated PCT levels occurred in severe shock, systemic inflammatory response syndrome and multiple organ dysfunction syndrome [29]. PCT was closely related to the occurrence and process of severe bacterial and septicemic infections, and could accurately reflect whether the source of infection causing lesions (such as peritonitis) has been eradicated [30]. Daily monitoring of PCT concentrations provided a reliable evaluation of treatment outcomes. PCT might be used to monitor surgical trauma or compound trauma.

The nomogram could be used to predict the risk of HIV positive patients and decided whether to undergo surgical treatment and preoperative interventions. The nomogram model helped doctors in treatment decisions. Compared with traditional tools (such as the NHSN index), the nomogram established in this study was more suitable for the assessment of infection risk of HIV positive patients after orthopedic surgery. Limited by the nature of the sample, this study was suitable for risk assessment of HIV positive patients after orthopedic surgery. In follow-up studies, we would include more samples and extend it to other populations.

However, there were some defects in the research. No a priori threshold was pre-specified, this might lead to a bias in the interpretation of the tests and generalisability of the results. Due to the nature of the sample, this study had certain limitations. In follow-up research, we would add some of the most common descriptive information to enhance the generality of the results. In a short period of time, no more samples could be collected, so in follow-up research, we would conduct external verification of the nomogram. The optimal cut-off point was calculated by Youden’s index method. And we would conduct external verification and include more indicators in follow-up research.

## Conclusion

In summary, a combination of CD4, ESR, and PCT might help researchers predict the incidence of SSI. Furthermore, the risk factors of SSI after clean implant orthopedic surgery in patients might provide the better evidence to guide the diagnosis and treatment of post-surgery SSI. The nomogram can be used to predict the risk of AIDS patients and decide whether to undergo surgical treatment and preoperative interventions. The nomogram model helps doctors in treatment decisions.

## Data Availability

The datasets used and analysed during the current study are available from the corresponding author on reasonable request.

## Abbreviations

SSI: Surgical site infection
OR: Odds ratio
ROC: Receiver Operating Characteristic
ESR: Erythrocyte sedimentation rate
PCT: Procalcitonin
AAOS: American Academy of Orthopaedic Surgeons
CRP: C-reactive protein
SD: Standard deviation
AUC: Area under curve
